# Downregulation of ASPP2 improves hepatocellular carcinoma cells survival via promoting BECN1-dependent autophagy initiation

**DOI:** 10.1038/cddis.2016.407

**Published:** 2016-12-08

**Authors:** Rui Chen, Hao Wang, Beibei Liang, Guoke Liu, Min Tang, Rongjie Jia, Xiaoyu Fan, Wei Jing, Xuyu Zhou, Huajing Wang, Yang Yang, Huafeng Wei, Bohua Li, Jian Zhao

**Affiliations:** 1 International Joint Cancer Institute, The Second Military Medical University, 800 Xiangyin Road, Shanghai 200433, People's Republic of China; 2 Shanghai University of Medicine and Health Sciences, 279 Zhouzhu Road, Shanghai 201318, People's Republic of China; 3 General Hospital of Lanzhou Military Command, 333 South Binhe Road, Lanzhou 730050, People's Republic of China; 4 Changhai Hospital, The Second Military Medical University, 168 Changhai Road, Shanghai 200433, People's Republic of China; 5 Cancer Center Key Lab, PLA General Hospital, 28 Fuxing Road, Beijing 100853, People's Republic of China

## Abstract

Autophagy is an important catabolic process, which sustains intracellular homeostasis and lengthens cell survival under stress. Here we identify the ankyrin-repeat-containing, SH3-domain-containing, and proline-rich region-containing protein 2 (ASPP2), a haploinsufficient tumor suppressor, as a molecular regulator of starvation-induced autophagy in hepatocellular carcinoma (HCC). ASPP2 expression is associated with an autophagic response upon nutrient deprivation and downregulation of ASPP2 facilitates autophagic flux, whereas overexpression of ASPP2 blocks this starvation-induced autophagy in HCC cells. Mechanistically, ASPP2 inhibits autophagy through regulating *BECN1* transcription and formation of phosphatidylinositol 3-kinase catalytic subunit type 3 (PIK3C3) complex. Firstly, ASPP2 inhibits p65/RelA-induced transcription of *BECN1*, directly by an ASPP2-p65/RelA-I*κ*B*α* complex which inhibits phosphorylation of I*κ*B*α* and the translocation of p65/RelA into the nucleus. Secondly, ASPP2 binds to BECN1, leading to decreased binding of PIK3C3 and UV radiation resistance-associated gene (UVRAG), and increased binding of Rubicon in PIK3C3 complex. Downregulation of ASPP2 enhances the pro-survival and chemoresistant property via autophagy in HCC cells *in vitro* and *in vivo*. Decreased ASPP2 expression was associated with increased BECN1 and poor survival in HCC patients. Therefore, ASPP2 is a key regulator of BECN1-dependent autophagy, and decreased ASPP2 may contribute to tumor progression and chemoresistance via promoting autophagy.

Autophagy is a lysosomal-dependent cellular degradation process, in which the cell self-digests its proteins and organelles and thus generates nutrients and energy to maintain essential cellular activities following nutrient starvation.^1^ Autophagy plays a critical role in the pathogenesis of diverse diseases, such as neuronal degeneration, aging, and cancer.^2, 3, 4, 5, 6^ There is increasing evidence demonstrating that autophagy is activated in cancer cells including hepatocellular carcinoma (HCC) under different stress conditions, such as starvation, growth factor deprivation, hypoxia, damaging stimuli, and therapeutic agents and such inducible autophagy constitutes an important pro-survival mechanism in response to cellular stresses.^7, 8^

*BECN1*, an important autophagy-related gene, is the first identified mammalian gene to induce autophagy.^9, 10^ It is a 60 kD protein containing a Bcl-2 homology domain (BH3), a coiled–coiled domain (CCD) and an evolutionarily conserved domain (ECD).^11, 12^ It is commonly expressed at very low levels in breast, prostate, and ovarian cancers;^9^ however, the expression of *BECN1* mRNA is significantly increased in liver tumor tissues and HCC cell lines despite that it is undetectable in normal liver tissues, indicating its important role in liver cancer survival.^13^ Mounting evidence indicates that one efficacious mechanism by which BECN1 promotes HCC cells survival is through autophagy induction.^14, 15, 16, 17^

The ankyrin-repeat-containing, SH3-domain-containing, and proline-rich region-containing protein (ASPP) family members are apoptosis regulation proteins, which consists of three members: ASPP1, ASPP2, and iASPP. ASPP1 and ASPP2 enhance, whereas iASPP inhibits, the activities of p53 and its family members p63 and p73. ASPP2 is a haploin-sufficient tumor suppressor, and aberrant expression of ASPP2 has been found in a variety of human cancers, including lung cancer, breast cancer, and leukemia.^18^ Our previous study also found that ASPP2 is downregulated by DNA methylation in HCC.^19^ Recent studies have also shown that ASPP2 inhibits RAS-induced autophagic activity to dictate the cellular response to RAS.^20^ However, it remains unknown whether downregulation of ASPP2 is involved in the regulation of autophagy in HCC. In this study, we provide evidence that downregulation of ASPP2 may contribute to tumor progression and chemoresistance via promoting BECN1-dependent autophagy in HCC.

## Results

### ASPP2 silencing is important for induction of autophagy in HCC

**Figure 1:**
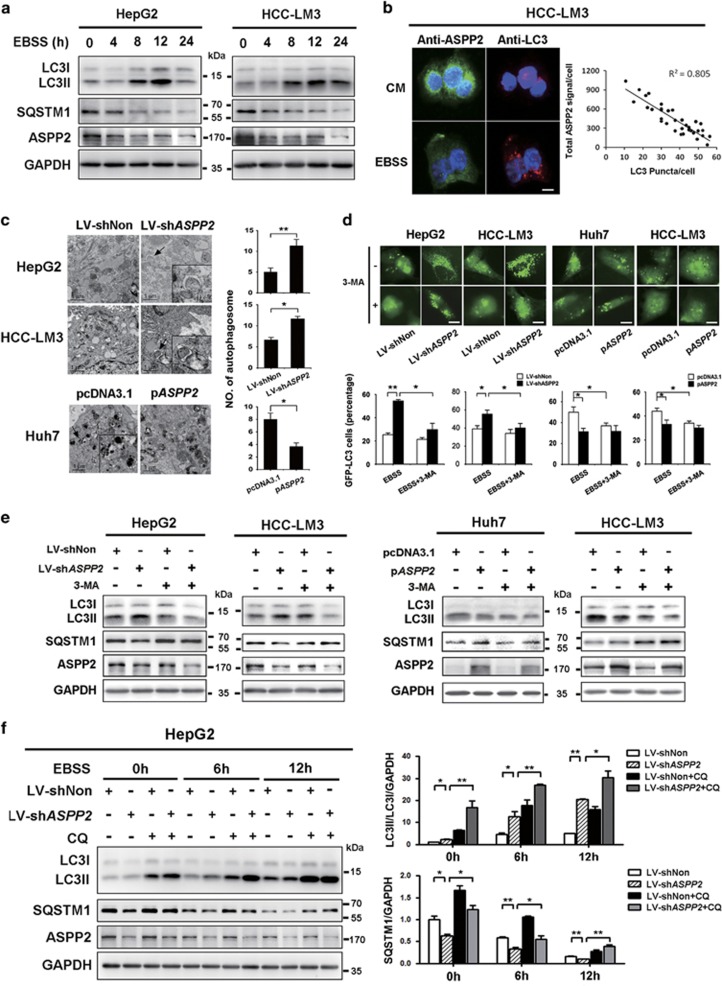
ASPP2 silencing is important for induction of autophagy in HCC cells. (**a**) HepG2 and HCC-LM3 liver cancer cells were incubate in CM (0 h) or EBSS for 4, 8, 12, and 24 h. Cells were collected for western blotting with antibodies. (**b**) HCC-LM3 cells were incubated in CM or in EBSS for 8 h. Cells were stained for ASPP2 or LC3 and imaged by immunofluorescence microscopy. Scale bars: 5 *μ*m. (**c** and **e**) HepG2 and HCC-LM3 were infected with LV-shNon or LV-sh*ASPP2* for 72 h; Huh7 and HCC-LM3 were transfected with pcDNA3.1 or p*ASPP*2 for 48 h. (**c**) Transmission electron microscopy showed formation of autophagosomes after EBSS treatment for 6 h in HCC cells. The scale bars represent 1 *μ*m. (**d**) Representative images of GFP-LC3 puncta (autophagosomes) in HCC cells cultured in EBSS for 6 h with or without 3-MA (10 mM, 1 h) pretreatment. Quantitation of autophagy (with GFP-LC3 punctate dots) in conditions shown in lower panel. The scale bars represent 10 *μ*m. (**e**) Western blots analysis of HCC cells cultured in EBSS for 6 h with or without 3-MA (10 mM, 1 h) pretreatment. (**f**) HepG2 infected with LV-shNon or LV-sh*ASPP2* were treated with EBSS for indicated time with or without CQ. Band intensity was quantified using ImageJ. Data represented the mean±S.D. from triplicate experiments. (*P<0.05; **P<0.01). CM, completed medium; EBSS, serum-free Earle's Balanced Salt Solution medium; 3-MA, 3-methyladenine; CQ, chloroquine

To determine the role of ASPP2 in the regulation of starvation-induced autophagy in HCC, we first tested the relationship between ASPP2 expression and nutrient deprivation in liver cancer cells. The protein levels of ASPP2 were significantly decreased upon Earle's Balanced Salt Solution medium (EBSS) treatment (Figure 1a and b). To measure the induction of autophagy during starvation, western blot analysis demonstrated conversion of LC3I to LC3II and degradation of SQSTM1/p62, a selective substrate that is degraded in autolysosomes, in a time-dependent manner after EBSS treatment. Decreasing ASPP2 protein level correlated with increased conversion of LC3I to LC3II in HepG2 and HCC-LM3 liver cancer cells following nutrient deprivation (Figure 1a). Consistent with these results, the starvation-induced increase in LC3 puncta also correlated with an decreased in ASPP2 protein in HCC-LM3 cells (Figure 1b).

We next tested the effect of silencing ASPP2 on nutrient deprivation-induced autophagy in HCC cells. After EBSS treatment, knockdown of *ASPP2* in HepG2 that with high levels of ASPP2 expression, and HCC-LM3 that with medium level of ASPP2 expression,^19^ increased cytoplasmic accumulation of autophagosomes and/or autolysosomes, as determined by the transmission electron microscopy (Figure 1c). In response to starvation, silencing *ASPP2* caused an increased accumulation of punctate GFP-LC3-positive autophagic vesicles, whereas these effects were attenuated by treatment with 3-methyladenine (3-MA) (Figure 1d). And the silencing of *ASPP2* increased the level of LC3II and decreased the expression of SQSTEM1/p62 (Figure 1e). These results were confirmed by the LC3 conversion and SQSTM1/p62 degradation experiments in HCC-LM3 cells by using two other LV-sh*ASPP2* (LV-sh*ASPP2*#2, LV-sh*ASPP2*#3) (Supplementary Figure 2A). In line with these results, overexpression of *ASPP2* significantly blocked starvation-induced autophagy in Huh7 that with low level of ASPP2 expression,^19^ and HCC-LM3 (Figure 1c and e). The effect of silencing and overexpression of ASPP2 in HCC cells was shown in Supplementary Figure 1.

Then, we examined changes in autophagic flux by comparing the levels of LC3II in the presence and absence of the lysosome inhibitor chloroquine (CQ). Treatment with LV-sh*ASPP2* significantly increased endogenous LC3II accumulation after starvation in HepG2 cells. Levels of LC3II were further increased by CQ-mediated inhibition of autolysome turnover, suggesting ASPP2 inhibited autophagic flux (Figure 1f). Consistent with the enhanced LC3 turnover, SQSTM1/p62 levels were significant decreased after ASPP2 silencing and prevented by CQ (Figure 1f). These results were further confirmed by the LC3 conversion and SQSTM1/p62 degradation experiments in HCC-LM3 cells (Supplementary Figure 2B). Overall, these results demonstrate that ASPP2 expression is associated with a robust autophagic response upon nutrient deprivation and downregulation of ASPP2 induces autophagic flux in HCC.

### Downregulation of ASPP2 promotes starvation-induced autophagy through regulating BECN1

**Figure 2:**
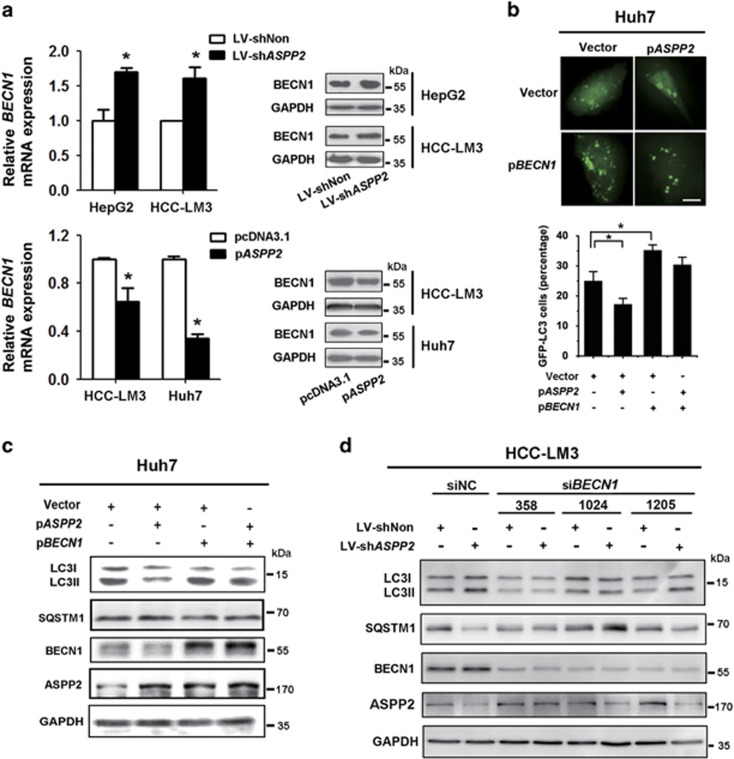
Downregulation of ASPP2 promotes starvation-induced autophagy through regulating BECN1. (**a**) HepG2 and HCC-LM3 cells were infected with LV-shNon or LV-sh*ASPP2* for 72 h; Huh7 and HCC-LM3 were transfected with pcDNA3.1 or p*ASPP*2 for 48 h. After cultured in EBSS, cells were performed for the analysis *BECN1* mRNA and protein level with quantitative PCR (left) and western blot (right). (**c**) Huh7 cells were co-transfected with p*EGFP-LC3* and p*BECN1* or p*ASPP2* into for 48 h, then were cultured in EBSS for 6 h. Representative images of GFP-LC3 (upper) and quantitation of the autophagic cells (lower). The scale bars represent 10 *μ*m. (**d**) Huh7 cells transfected and treated as (**c**), but without p*EGFP-LC3* were lysed and subjected to western blotting. (**d**) HCC-LM3 cells infected with LV-shNon or LV-sh*ASPP2* for 72 h were transfected with si*BECN1* for 48 h, and then were incubated in EBSS for 6 h. Cells lysates were subjected to western blotting. Data represented the mean±S.D. from triplicate experiments. (*P<0.05). 3-MA, 3-methyladenine

To further explore the autophagic regulation by ASPP2, we then detected the mRNA levels of *ATG5*, *ATG7*, and *BECN1*, which were well-documented autophagy-related proteins. Quantitative real-time PCR showed that the mRNA levels of *ATG5*, *ATG7*, and *BECN1* were higher in *ASPP2*-silenced HepG2 and HCC-LM3, which can be rescued by overexpressing *ASPP2* in Huh7 and HCC-LM3 (Figure 2a and Supplementary Figure 3). Consistent with the mRNA results, silencing of *ASPP2* increased the expression of BECN1 on protein level while overexpression of *ASPP2* suppressed BECN1 (Figure 2a).

To confirm the role of BECN1 in ASPP2-regulated autophagy, p*BECN1* and p*ASPP2* were co-transfected into Huh7. Overexpression of BECN1 in Huh7 reversed the decreased accumulation of punctate GFP-LC3-positive autophagic vesicles and decreased conversion of LC3I to LC3II and degradation of SQSTM1/p62 induced by ASPP2 overexpression upon nutrient withdrawal (Figure 2b and c). In addition, knockdown of BECN1 with three different siRNA in HCC-LM3 inhibited the promotion of autophagy by silencing ASPP2 (Figure 2d). These results suggest that decreased ASPP2 might promote autophagic process through regulating BECN1.

### Downregulation of ASPP2 promotes p65/RelA-dependent transactivation of *BECN1*

**Figure 3:**
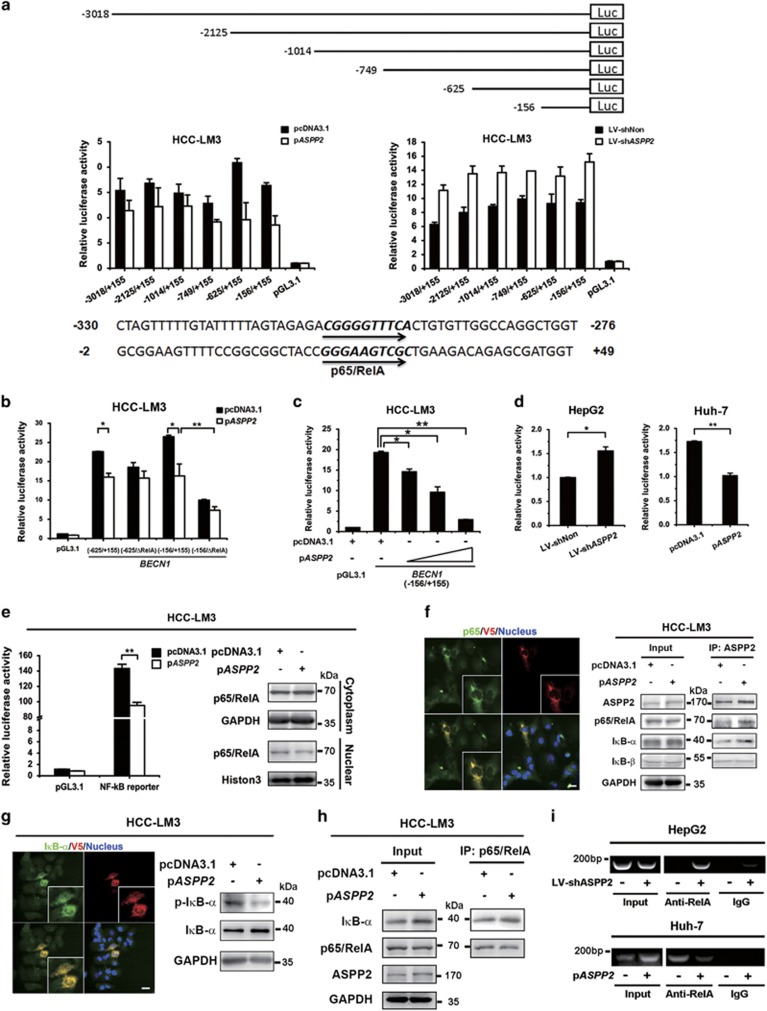
ASPP2 inhibits NF-*κ*B/p65-dependent transactivation of *BECN1*. (**a**) Various truncated BECN1 promoters were generated by PCR and inserted into reporter vector pGL3.1 (upper). These constructs were co-transfected with an internal control vector into HCC-LM3 cells with overexpression or silencing of ASPP2. The cells were collected for analysis of luciferase activities (middle). The JASPAR database was queried for consensus binding sites of known transcription factors for the sequence nt −305 to −296 and nt +22 to +31 of the *BECN1* promoter (lower). (**b**) HCC-LM3 cells transfected with vector or p*ASPP2* were transfected with *BECN1* (−625/+155) and (−156/+155)-luc or the promoter containing p65/RelA consensus binding site mutation (cggggtttca→aaattgaaga, *BECN1*-625/ΔRelA) and (gggaagtcgc→aagatgaagc, *BECN1*-156/ΔRelA), and luciferase activities were measured at 48 h posttransfection followed by 6 h EBSS treatment. (**c**) HCC-LM3 cells were co-transfected with *BECN1* (−156/+155)-luc and increasing amounts of ASPP2-expressing plasmids (0.5, 1.0, and 1.5 *versus* control). (**d**) HepG2 cell infected with LV-shNon or LV-sh*ASPP2* were transfected with *BECN1* (−156/+155)-luc, and Huh7 cell lines were co-transfected with *BECN1* (−156/+155)-luc and p*ASPP2* (right). (**e**) HCC-LM3 cells were co-transfected with vector or p*ASPP2* and NF-*κ*B-driven luciferase construct (left). HCC-LM3 cells transfected as indicated, the nuclear and cytoplasm extracts were immunoblotted with the indicated antibodies (right). (**f**) HCC-LM3 cells transfected with p*ASPP2*-V5 were stained with anti-p65/RelA (green) and anti-V5 (red) antibodies and imaged by IF. Scale bars: 30 *μ*m (left). The ASPP2-p65/RelA and ASPP2-I*κ*B*α* conjugates were immunoprecipitated with anti-ASPP2 and analyzed by western blotting (right). (**g**) HCC-LM3 cells transfected with p*ASPP2*-V5 were stained with anti-V5 (red) and anti-I*κ*B*α* (green) antibodies. Scale bars: 30 *μ*m (left). Cell lysates were subjected to western blotting (right). (**h**) The I*κ*B*α*-p65/RelA complex was immunoprecipitated with anti-p65/RelA and analyzed by western blotting. (**i**) Chromatin-immunoprecipitation analysis was performed on HepG2 and Huh7 cell lysates using antibodies against p65/RelA. Data are shown as the means±S.D. from triplicate experiments. (*P<0.05; **P<0.01)

There was a negative correlation between ASPP2 and BECN1 mRNA (Figure 2a), suggesting that ASPP2 might inhibit BECN1 transcription during autophagy. To investigate the potential mechanism, the transcriptional activities of various truncated mutants of *BECN1* promoter (5′-flanking region) were measured in HCC-LM3 cells (Figure 3a, upper). The luciferase reporter assay indicated that the *BECN1* (−625/+155) exhibited the higher luciferase activity. With overexpression of ASPP2, we also found that ASPP2 greatly inhibited these *BECN1* promoter activities, among which *BECN1* (−625/+155) and (−156/+155) had much higher promoter activity (Figure 3a, middle). Consistently, silencing of ASPP2 enhanced *BECN1* promoter activities, especially in the truncated mutants (−625/+155) and (−156/+155) (Figure 3a, middle). Thus, ASPP2 might target nt −625 to nt +155 *cis*-regulatory elements to repress *BECN1* transcription.

To predict the nuclear factors that bind to this *cis*-element of interest, JASPAR database was used to analyze the nucleotide sequence of the *BECN1* promoter region nt −625 to nt +155. We found that the regions nt −305 to −296 and nt +22 to +31 matched the known consensus binding site of p65/RelA (Figure 3a, lower). p65/RelA was reported to positively modulates autophagy through regulating *BECN1* transcription.^21^ Moreover, p65/RelA is involved in the regulation of apoptosis induced by ASPP2.^22^ Thus, we hypothesized that ASPP2 might inhibit *BECN1* promoter activity through attenuating the activity of p65/RelA. Then new luciferase reporter plasmids of *BECN1* promoter (−625/+155) and (−156/+155) contained mutations at the p65/RelA binding sites were constructed. Mutation can significantly attenuate *BECN1* promoter transcription activity both in control and ASPP2-overexpressed or ASPP2-silenced cells at the site nt +22 to +31, not at nt −305 to −296 (Figure 3b and Supplementary Figure 4A). The results suggested that p65/RelA could bind the site at nt +22 to +31 to regulate transcription of *BECN1*, and ASPP2 would inhibit this process. A dose-dependent relationship was examined in the repression of *BECN1* (−156/+155)-luciferase activities by ASPP2 expression plasmid (Figure 3c). Similar results regarding the BECN1 promoter (−156/+155) activities regulated by ASPP2 were further confirmed in HepG2 and Huh7 (Figure 3d).

To further assess the potential association between ASPP2 and p65/RelA, NF-*κ*B reporter plasmid was used to examine the NF-*κ*B activity, which was suppressed by overexpression of ASPP2 and enhanced by knockdown of ASPP2 in HCC-LM3 cells (Figure 3e, left and Supplementary Figure 4B). The NF-*κ*B pathway is activated through active p65/p50 complex, which needs to be released from I*κ*B*α* and translocates into the nucleus.^23^ We found that overexpression of ASPP2 inhibited the expression of p65/RelA in the nucleus, whereas inhibition of ASPP2 expression enhanced p65/RelA entry into the nucleus (Figure 3e, right and Supplementary Figure 4C).

As ASPP2 is a known binding protein of p65/RelA,^22^ we tested its ability to repress the entry of p65/RelA into the nucleus by binding and inactivating p65/RelA. Double IF staining showed that endogenous p65/RelA co-localized with ASPP2 in the cytoplasm of HCC-LM3 cells (Supplementary Figure 4D). Exogenously expressed cytoplasmic ASPP2 also co-localized with p65/RelA, retaining more p65/RelA in the cytoplasm (Figure 3f, left). ASPP2 co-immunoprecipitated with p65/RelA, I*κ*B*α*, and I*κ*B*β* in HCC-LM3 cells. The amount of p65/RelA and IkB*α* bound with ASPP2 was increased in ASPP2-overexpressed cells, and decreased in ASPP2-silenced cells (Figure 3f, right and Supplementary Figure 4E). These results indicate that ASPP2 binds to p65 and I*κ*B*α*. Furthermore, the interaction between ASPP2 and I*κ*B*α* inhibited phosphorylation of I*κ*B*α*, inducing cytoplasmic accumulation of I*κ*B*α* (Figure 3g). In contrast, downregulation of ASPP2 promotes phosphorylation of I*κ*B*α* in HCC-LM3 cells under starvation environment (Supplementary Figure 4E). The amount of the IκB*α* bound with p65/RelA was visibly higher after overexpression of ASPP2 in HCC-LM3 cells (Figure 3h).

To confirm the intracellular effect of p65/RelA binding to *BECN1* promoter, chromatin-immunoprecipitation was performed in HepG2 and Huh7 cells to monitor the recruitment of p65/RelA to the BECN1 promoter. Chromatin-immunoprecipitation analysis revealed that overexpression of ASPP2 in Huh7 cells resulted in less DNA of the BECN1 promoter co-immunoprecipated with p65/RelA. In contrast, downregulation of ASPP2 in HepG2 cells resulted in the recruitment of more p65/RelA on the BECN1 promoter (Figure 3i). These results indicate that ASPP2 negatively regulates the expression of BECN1 by abrogating p65/RelA-dependent transcription in HCC cells. We also detected the effect of p65/RelA in autophagy regulated by ASPP2. We found that overexpression of BECN1 or p65/RelA with ASPP2 in HCC-LM3 cells increased the conversion of LC3I to LC3II and the degradation of p62/SQSTEM1 compared with only overexpression ASPP2 (Supplementary Figure 4G). These results suggested that p65/RelA reversed ASPP2-impaired autophagy.

### ASPP2 inhibits autophagy via interaction with BECN1

A proteomic analysis predicted that TP53BP2 (ASPP2) interacted with BECN1.^24^ As BECN1 is a key component of phosphatidylinositol 3-kinase catalytic subunit type 3 (PIK3C3) complex, we asked whether the interaction between ASPP2 and BECN1 was involved in the regulation of this autophagy initiation complex.

**Figure 4:**
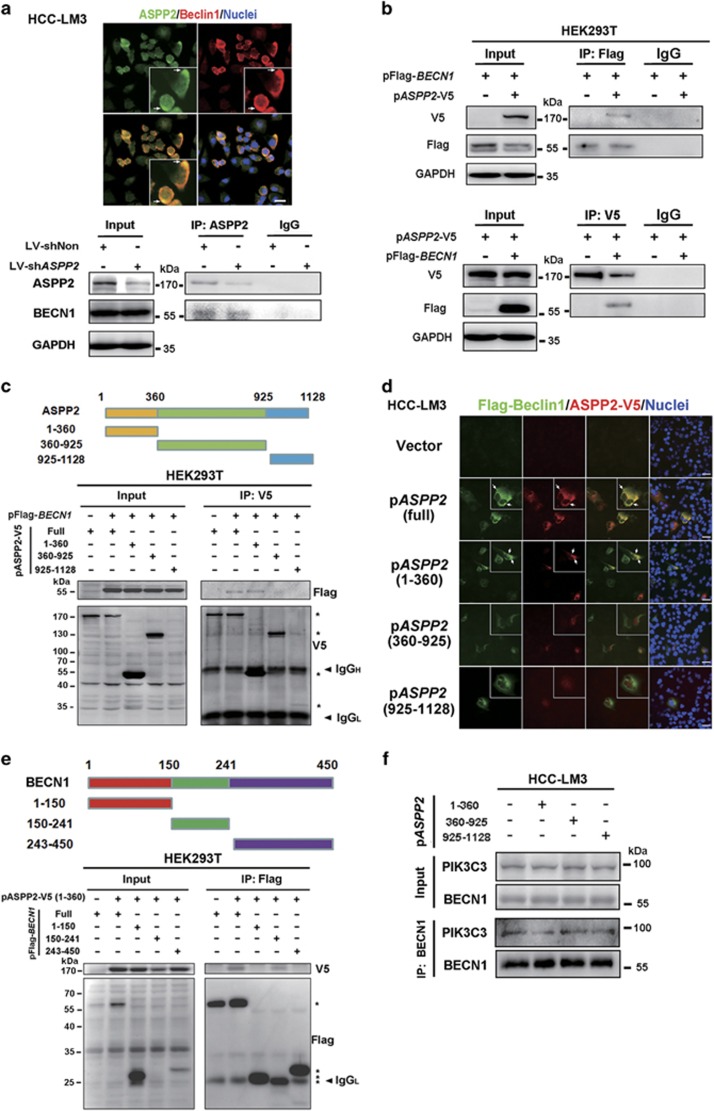
ASPP2 colocalizes with BECN1 and co-immunoprecipitates with BECN1. (**a**) HCC-LM3 were treated with EBSS for 6 h. Location of endogenous ASPP2 and BECN1 was analyzed by double IF. Scale bars: 30 *μ*m (upper). The ASPP2-BENC1 complex was immunoprecipitated with anti-ASPP2 and analyzed by western blotting (lower). (**b**) Flag-tagged BECN1 was co-transfected with ASPP2-V5 into HEK293T cells, which were analyzed by western blotting following IP with anti-Flag antibody (upper). V5-tagged ASPP2 was co-transfected with Flag-BECN1 into HEK293T cells, which were analyzed by western blotting following IP with anti-V5 antibody (lower). (**c**) Schematic representation of truncated ASPP2 mutants (upper). V5-tagged full-length and deletion mutants of ASPP2 were co-transfected with Flag-BECN1 into HEK293T. V5 antibody was used to immunoprecipitate full-length and fragments of ASPP2 (*, V5-tagged protein). (**d**) Flag-BECN1 and V5-tagged ASPP2 fragments were co-transfected into HCC-LM3 cells for 48 h, followed by 6 h EBSS treatment. Anti-V5 (red) and anti-flag (green) antibodies were used to analyze location of proteins by double IF staining. Scale bars: 30 *μ*m. (**e**) Schematic representation of truncated BECN1 mutants (upper). Flag-tagged full-length and deletion mutants of BECN1 were co-transfected with ASPP2-V5(1-360) into HEK293T. Flag antibody was used to immunoprecipitate full-length and fragments of BECN1 (*, Flag-tagged protein). (**f**) ASPP2 fragments were transfected into HCC-LM3 cells for 48 h, followed by 6 h EBSS treatment. Endogenous the PIK3C3 complexes were immunoprecipitated with anti-BECN1 antibody and analyzed for co-immunoprecipitation of BECN1-PIK3C3 conjugates (IP). Twenty-four hours after transfection, HEK293T cells were treated with EBSS for 6 h in (**b**), (**c**), and (**e**). In all IP analyses, host species-matched nonspecific IgG served as negative controls, and whole-cell lysates (input) are included for comparison

In immunofluorescent staining, BECN1 co-localized with endogenous ASPP2 in part of the cells during starvation-induced autophagy (Figure 3a, upper). Endogenous ASPP2 was found to co-immunoprecipitate with endogenous BECN1 in HCC-LM3 during growth in EBSS medium, and the interaction was reduced when ASPP2 was downregulated (Figure 4a, lower). This interaction was further confirmed by ectopic expression of V5-tagged ASPP2 and flag-tagged BECN1 in HEK293T cells (Figure 4b).

To identify the interaction domains between ASPP2 and BECN1, three ASPP2 mutants were used: ASPP2(1-360), ASPP2(360-925), and ASPP2(925-1128). Each V5-tagged ASPP2 mutants was tested for binding with flag-tagged BECN1 in HEK293T cells. ASPP2(1-360), but not ASPP2(360-925) and ASPP2(925-1128) was able to co-immunoprecipitate with BECN1 (Figure 4c). Similar result was observed in HCC-LM3 cells by immunofluorescent staining using transfected flag-tagged BECN1 and V5-tagged ASPP2 fragments (Figure 4d). These data suggested that N-terminal ASPP2 binds BECN1. We also used a series of flag-tagged BECN1 deletion mutants (BECN1[1–150], BECN1[150–241], and BECN1[243–450]) and examined their interaction with V5-tagged ASPP2(1-360). The middle domain of BECN1 (aa, 150–241, CCD domain), not the N terminus and C terminus, was required for interaction with ASPP2 (Figure 4e). These results suggested that the interaction between ASPP2 and BECN1 might influence the function of BECN1 CCD domain.

**Figure 5:**
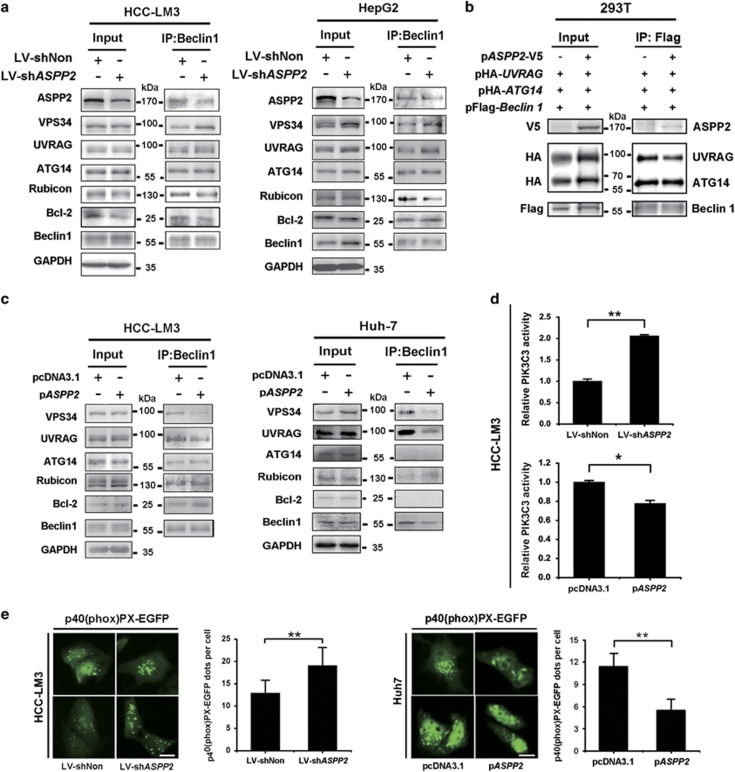
Downregulation of ASPP2 increases the BECN1 interactome and BECN1-associated PIK3C3 kinase activity in HCC cells. (**a**) HCC-LM3 and HepG2 infected with LV-shNon or LV-sh*ASPP2*, followed by 6 h EBSS treatment, were lysed for immunoprecipitation with BECN1-specific antibody and immunoblotted with antibodies as indicated. (**b**) HEK293T cells were transfected with Flag-BECN1, HA-UVRAG, HA-ATG14, and ASPP2-V5 or Flag-BECN1, HA-UVRAG, HA-ATG14, and V5-vector for 24 h, and then treated with EBSS for 6 h. Cell lysates were immunoprecipitated with Flag antibody (BECN1) and analyzed by western blotting. (**c**) HCC-LM3 and Huh7 transfected with pcDNA3.1 or p*ASPP2*, followed by 6 h EBSS treatment, were lysed for immunoprecipitation with BECN1-specific antibody and immunoblotted with antibodies as indicated. (**d**) HCC-LM3 cells infected with LV-shNon or LV-sh*ASPP2* for 72 h, or transfected with vector or p*ASPP2* for 48 h, were treated with EBSS for 6 h. Then cell lysates were immunoprecipitated with anti-BECN1 antibody. The PI3-Kinase activity ELISA kit was used to measure the kinase activity of the PIK3C3 protein immunoprecipitated. (**e**) At 24 h posttransfection with p40(phox)PX-EGFP fusion, HCC-LM3 with LV-shNon or LV-sh*ASPP2* were detected using an inverted fluorescence microscope. Huh7 were detected after co-transfection with p40(phox)PX-EGFP fusion and p*ASPP2* vectors for 48 h. p40(phox)PX-EGFP-positive vesicles were quantified and analyzed. The scale bars represent 10 *μ*m. Data are shown as the means±S.D. from triplicate experiments. (*P<0.05; **P<0.01)

Formation of the PIK3C3 complex plays an essential role in autophagy.^11^ We next examined the role of ASPP2 in forming the PIK3C3 complex and the ability of ASPP2 to regulate PIK3C3 kinase activity. We observed that the BECN1–PIK3C3 interaction was increased in HCC-LM3 and HepG2 cells with downregulated ASPP2 during starvation-induced autophagy (Figure 5a). Moreover, only ASPP2(1-360), the domain of ASPP2 binding to BECN1, could inhibit interaction between BECN1 and PIK3C3 (Figure 4f). We also examined the effects of downregulated ASPP2 on the interaction between BECN1 and its binding partners, BCL2, Rubicon, PIK3C3, UV radiation resistance-associated gene (UVRAG), and ATG14 in HCC-LM3 and HepG2 cells. Rubicon, which are negative regulator of PIK3C3 kinase activity, were decreased by co-immunoprecipitation in ASPP2-silencing cells, but not BCL2. As the promoters of PIK3C3 kinase activity, only UVRAG bound with BECN1 was increased in ASPP2-silenced cells. ATG14 did not change obviously (Figure 5a). UVRAG and ATG14 could bind to the BECN1 CCD domain, which also binds to ASPP2 from our above results (Figure 4e). Thus, we tested whether ASPP2 could disrupt BECN1 interaction with UVRAG rather than ATG14 by co-immunoprecipiation with BECN1. As expected, overexpression of ASPP2 induced less level of UVRAG, but not ATG14, bound to BECN1 (Figure 5b). These results were confirmed in HCC-LM3 and Huh7 cells with overexpression of ASPP2 (Figure 5c). All of these indicated that binding of ASPP2 to BECN1 suppresses formation of the PIK3C3 complex by disrupting the interaction between BECN1 and UVRAG.

The BECN1–PIK3C3 interaction is associated with increased PIK3C3 kinase activity. Overexpression of ASPP2 decreased PIK3C3 kinase activity, and silencing of ASPP2 increased it (Figure 5d). The analysis of foci formation of p40(phox)PX-EGFP fusion protein demonstrated that downregulation of ASPP2 significantly increased PIK3C3 lipid kinase activity in HCC-LM3 cells (Figure 5e). Conversely, overexpression of ASPP2 in Huh7 decreased the levels of BECN1–PIK3C3 interaction and inhibited the lipid kinase activity of PIK3C3 during autophagy (Figure 5e). All these results suggest that binding of ASPP2 to BECN1 can suppress or destabilize the interaction of the BECN1, PIK3C3, and UVRAG core complex, contributing to the decreased PIK3C3 kinase activity.

### Autophagy enhanced by downregulation of ASPP2 contributes to survival and chemoresistance of HCC

**Figure 6:**
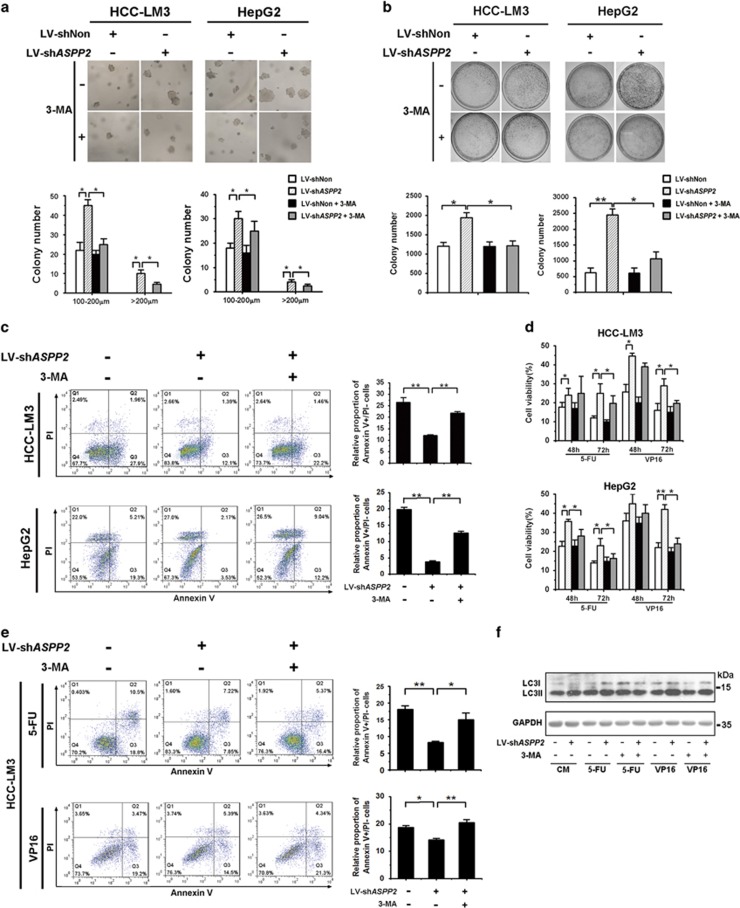
Autophagy enhanced by downregulation of ASPP2 contributes to survival and chemoresistance of HCC cells. (**a**) HCC-LM3 and HepG2 cells infected with LV-shNon or LV-sh*ASPP2* were treated with EBSS for 6 h with or without 3-MA pretreated 1 h, and then plated in semisolid soft agar medium to monitor anchorage-independent growth. (**b**) The cells handled as above were plated in dishes for colony assay. (**c**) HCC-LM3 and HepG2 cells infected with LV-shNon or LV-sh*ASPP2* were pretreated with 10 mM 3-MA or not for 1 h followed by culture in EBSS for 24 h. The percentage of apoptotic cells was measured by Annexin V-FITC/PI (propidium iodide) double staining. The early apoptotic cells (Annexin V+/PI-) were quantified. (**d**) The infected HCC cells pretreated with 3-MA for 1 h were seeded in 96-well plates with 5-FU (7.5 *μ*g/ml) or VP16 (30 *μ*g/ml) for MTS assay at 48 and 72 h. Values were given as the means±S.D. of six wells. (**e**) The cells handled as (**d**) for 24 h were analyzed by Annexin V-FITC/PI double staining for apoptosis. The early apoptotic cells (Annexin V+/PI-) were quantified. (**f**) The cells handled as (**e**) were analyzed by western blotting for conversion of LC3I to LC3II. Data are shown as the means±S.D. from triplicate experiments. (*P<0.05; **P<0.01)

To further investigate the role of ASPP2 in tumor development through regulating autophagy, we analyzed cell proliferation and survival during nutrition deprivation. The anchorage-independent cell growth of HepG2 and HCC-LM3 were significantly enhanced by ASPP2 silencing, and the colony foci greater than 200 *μ*m were found in ASPP2 silencing groups (Figure 6a). These changes by knockdown of ASPP2 can be compromised by 3-MA, indicating that the ASPP2 downregulation increases the development of HCC by facilitating starvation-induced autophagy. The results of plate-colony assay showed the same tendency (Figure 6b). It has been reported that autophagy activation elicited by starvation serves as a pro-survival mechanism. Then, apoptosis was investigated in ASPP2-silenced cells. Knockdown of ASPP2 attenuated starvation-induced apoptosis in HepG2 and HCC-LM3, and 3-MA can compromise these effects (Figure 6c).

Current evidence supports the idea that tumor resistance to anticancer therapies including chemotherapy can be enhanced through upregulation of autophagy in various tumor cell lines.^25^ MTS (3-(4,5-dimethylthiazol-2-yl)-5-(3-carboxymethoxyphenyl)-2-(4-sulfophenyl)-2H-tetrazolium, inner salt) assay showed that knockdown of ASPP2 increased tumor cell viability under the treatment of fluorouracil (5-FU) or etoposide (VP16), whereas inhibition of autophagy by 3-MA could block the autophagy-induced chemoresistance (Figure 6d). Meanwhile, increased percentage of apoptotic cells in ASPP2-silenced cell with 5-FU or VP16 with 3-MA treatment, compared with only 5-FU or VP16-treated cells (Figure 6e). After downregulation of ASPP2, the conversion of LC3I to LC3II induced by chemotherapeutic agent were increased in HCC-LM3 cells (Figure 6f).

**Figure 7:**
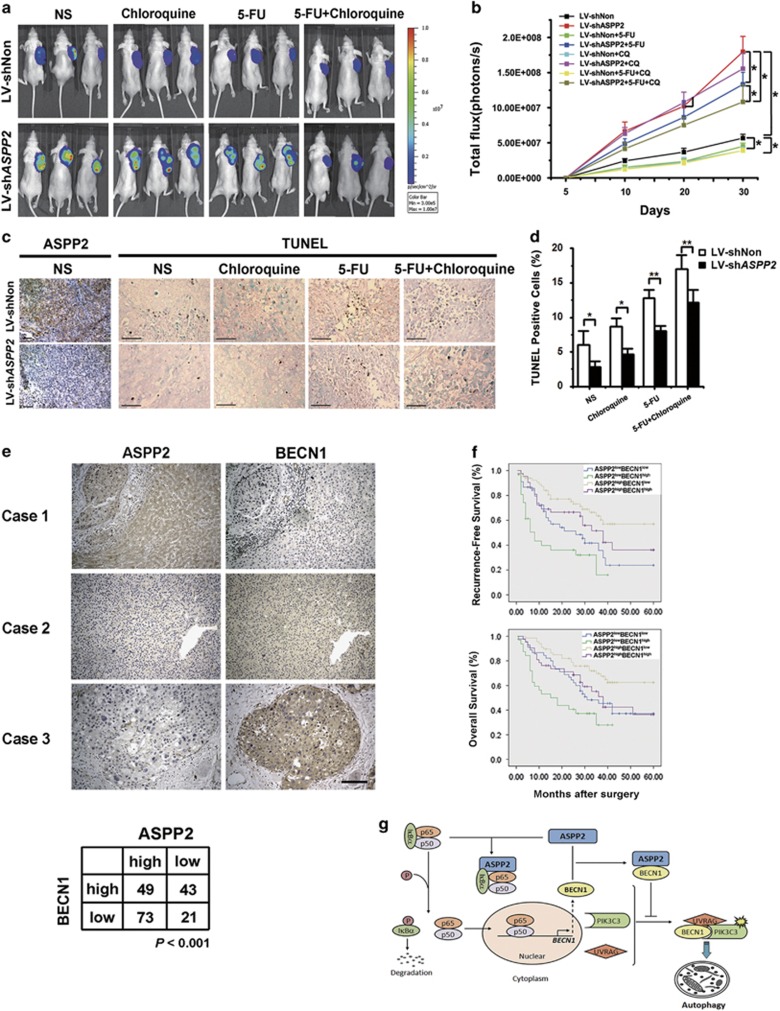
(**a**–**e**) Inhibition of autophagy blocked chemoresistance of HCC xenografts induced by downregulation of ASPP2 in nude mice. (**a**) Whole-body bioluminescent images of HCC-LM3-luc tumor xenograft mice were determined by the Xenogen IVIS 100 imaging System. (**b**) Quantitate fluorescence intensities of tumors from *in vivo* images at at indicated time points. The fluorescence intensity of the mice was measured by recording CCD photon counts. (**c**) TUNEL analysis was performed in tumor sections derived from the mice as in (**a**) at day 30 after cell inoculation. The apoptotic nuclei are seen as dark brown color under a microscope. Cell nuclei were counter stained with methyl green. Representative images of ASPP2 immunostaining in xenograft tumors (left). Scale bars represent 40 *μ*m. (**d**) The percentage of apoptotic cells was calculated by counting brown-stained nuclei *versus* green-stained nuclei from five randomly chosen fields in each section. Data are present as means±S.D. (*P<0.05; **P<0.01) (**e**–**f**) ASPP2 and BECN1 expression in human HCC tissues. (**e**) Representative images of ASPP2 and BECN1 expression in HCC tissues examined by immunohistochemistry. Scale bars represent 100 *μ*m. The statistical correlation between ASPP2 and BECN1 expression are shown on lower panel. (**f**) Kaplan–Meier analysis of survival in patients with HCC. Kaplan–Meier survival curves for patients with HCC according to levels of ASPP2 and BECN1 expression were shown in upper (RFS) and lower (OS). (**g**) Diagram summarizing the role of ASPP2 in regulating BECN1-dependant autophagic activity. CQ, chloroquine

To further confirm the autophagy-dependent promotion of tumor growth and chemoresistant effect of ASPP2 downregulation, luciferase-expressing HCC-LM3-luc cells infected with LV-sh*ASPP2* or LV-shNon were injected into the flank of nude mice, which were treated with or without CQ and 5-FU. Thirty days after xenografting, downregulation of ASPP2 significantly enhanced the growth of HCC-LM3 cells in nude mice, though 5-FU decreased the rate of tumor growth partly. However, CQ can delay tumor growth in the ASPP2 silenced and control group, especially combined with 5-FU (Figure 7a and b). Consistent with the in *vitro* experimental results, fewer apoptotic cells were found in HCC-LM3 xenografts with ASPP2 silencing, and CQ overcame this chemoresistance, resulting in activation of apoptosis (Figure 7c and d). All these data demonstrate that silencing of ASPP2 can enhance the ability of resistance to chemotherapeutic agent through autophagy activation in HCC.

### Expression of ASPP2 correlates negatively with BECN1 in surgical specimens of HCC

To assess the clinical relevance of ASPP2 and BECN1, we further examined ASPP2 and BECN1 protein expression in 186 HCC tissues by immunohistochemistry (Figure 7e). There was no significant correlation between ASPP2 expression and age, gender, alpha-fetoprotein level, and tumor size. A total of 59.8% (73/122) of the tumor samples in which ASPP2 had high expression, showed low expression of BECN1, whereas high expression of BECN1 was found in about 67.2% (43/64) of tumor samples in which ASPP2 was low expression. The correlation between ASPP2 and BECN1 expression was inversely associated (*P*<0.001; Figure 7e). The results further supported our conclusion that ASPP2 negatively regulated BECN1 expression in HCC.

The correlation in clinic pathologic parameters in all HCC patients was statistically analyzed (Supplementary Table S1). A significant correlation between the BECN1 expression and tumor volume was observed (*P*=0.002). Importantly, in the ASPP2 low group, a significant correlation between the presence of BECN1 and advanced tumor volume was observed (*P*=0.008). In contrast, this correlation was not statistically significant in the ASPP2 high group (*P*=0.166; Supplementary Table S2). No significant correlation was found between BECN1 expression and other variables, including age, sex, tumor stage, or recurrence time. The potential association between ASPP2 or BECN1 expression level and recurrence-free survival (RFS) or overall survival (OS) was retrospectively evaluated. Kaplan–Meier analysis showed that RFS and OS (Figure 7f) were significantly worse among patients in ASPP2^low^/BECN1^high^ group.

In univariate analysis, ASPP2 and BECN1 expression status were prognositic factors for RFS and OS (Supplementary Table S3). The tumor number was significantly associated with OS and AJCC stage with RFS. ASPP2 and BECN1 were prognostic for RFS (*P*<0.001) and OS (*P*<0.001, Supplementary Table S3). In multivariate analysis, tumor number, ASPP2, and BECN1 expression status remained the significant independent predictors of RFS and OS. Patients with high expression of BECN1 were about 1.669 times more likely to suffer from relapse than ones with low expression of BECN1 (hazard ratio: 1.669; 95% confidence interval: 1.090–2.555). Patients with high expression of ASPP2 were about 0.623 times less to suffer from relapse than ones with low expression of ASPP2 (hazard ratio: 0.623; 95% confidence interval: 0.405–0.960). AJCC stage was the independent predictor only for RFS (Supplementary Table S4). Thus, increased expression of BECN1 with decreased ASPP2 may serve as a prognostic indicator for patients with HCC.

## Discussion

Autophagy plays a critical role in the pathogenesis of diverse disease, such as neuronal degeneration, aging, and cancer.^26^ Here we demonstrate that downregulation of ASPP2 promotes the development of HCC by enhancing starvation-induced autophagy via regulating regulating *BECN1* transcription and formation of PIK3C3 complex (Figure 7g).

In our study, the trends of BECN1 change are the most consistent in ASPP2-silencing and ASPP2-overexpression HCC cells. Although ATG5/7 are almost influenced by ASPP2 in HepG2 and Huh7, the change levels of ATG5/7 are not significant in HCC-LM3 cells with ASPP2 overexpression (Supplementary Figure 3). Meanwhile, there is no report about the correlation between ASPP2 and BECN1 in autophagy. Therefore, we focused on the regulation of ASPP2 on BECN1 in this study.

In mechanism, we found that ASPP2 can bind p65/RelA, preventing p65/RelA activating *BECN1* transcription. Recently, ASPP2 was found to inhibit *Δ*Np63 expression through its ability to bind I*κ*B*β* and enhance nuclear p65/RelA, which mediates the repression of p63 in squamous cell carcinoma cells.^27^ In this study, however, we showed that overexpression of ASPP2 did not increase I*κ*B*β* bound with ASPP2 in HCC cells during starvation-induced autophagy (Figure 3f), suggesting the level of I*κ*B*β* expression might be limited and not the key regulator of p65/RelA in HCC cells or under nutrient-deprived environment. Instead of I*κ*B*β*, we found that ASPP2 interacted with p65/RelA and I*κ*B*α*, maintaining the p65/RelA-I*κ*B*α* complex and suppressing phosphorylation of I*κ*B*α*, which can trigger itself degradation and release p65/RelA.^23^

Second, two stable PIK3C3 complexes have been described in both yeast and mammals are implicated in autophagosome formation.^11^ Complex II (containing UVRAG) follows the stage of complex I (containing ATG14) regulation. In complex II, UVRAG bridges BECN1 and PIK3C3 complex.^28^ Our data show that ASPP2 disrupts the association of complex II rather than the formation of complex I. Interestingly, downregulation of ASPP2 inhibits combination of BECN1 and rubicon, which is a suppressor of the PIK3C3 complex. Rubicon binds only to a subpopulation of UVRAG complexes.^29^ These data further proved that ASPP2 specifically regulates the function of complex II during autophagosome formation. Recently, ASPP2 was reported to induce autophagic apoptosis by decreasing BCL2 expression and maintaining nuclear ASPP2-BCL2 complexes.^30^ It focused on the role of nuclear ASPP2 on autophagy. However, our study focuses on the function of ASPP2 in cytoplasm on autophagy. Moreover, ASPP2 binds BECN1 at its CCD domain, which is not associated with BCL2. Therefore, the function of cytoplasmic ASPP2 on autophagy might be different from the one of nuclear ASPP2.

## Materials and Methods

### Cell culture

HepG2 and HEK293T cells were purchased from American Type Culture Collection (ATCC). HCC-LM3 was obtained from the Liver Cancer Institute, Zhong Shan Hospital, Fudan University (Shanghai, People's Republic of China). Huh7 was obtained from Cell Bank of Shanghai Institutes for Biological Sciences, Chinese Academy of Sciences (Shanghai, People's Republic of China). A polyclonal population of HCC-LM3 stably expressing the Luc reporter gene was generated by transfection with pcDNA-Luc plasmid and a Geneticin (G418) selection. All these cell lines were cultured in DMEM (Gibco, Waltham, MA, USA) supplemented with 10% (vol/vol) FBS (Gibco), 100 U/ml penicillin and 100 *μ*g/ml streptomycin (Invitrogen, Waltham, MA, USA) at 37 °C in a humidified incubator containing 5% CO_2_. For serum and amino acid starvation, cells were cultured in serum-free EBSS (Sigma-Aldrich, Darmstadt, Germany, E2888), which we refer to as nutrient-free medium. 3-methyladenine (3-MA; 10 mM; Sigma-Aldrich, M9281), chloroquine (CQ; 30 *μ*M; Sigma-Aldrich, C6628).

### Plasmids, small interfering RNA, and lentivirus

pcDNA3.1-*ASPP2* (full, (1–360), (360–925), (925–1128))-V5 was obtained from Dr Xin Lu's Lab at Ludwig Institute for Cancer Research, Oxford, UK. pcDNA3.0-flag-*BECN1* was obtained from Prof. Mujun Zhao's Lab at Institute of Biochemistry and Cell Biology, Chinese Academy of Science, Shanghai, People's Republic of China. pCMV-*p65/RelA*-flag was constructed by PCR and inserted into pCMV-C-Flag vector (Beyotime Biotech. D2632, Shanghai, People's Republic of China). Various truncated BECN1 promoters were generated by PCR and inserted into reporter vector pGL3.0-enhancer (Promega, Madison, WI, USA). p40(phox)PX-EGFP, pFlag-*BECN1* ((1–150), (150–241), (242–450)), pCI-neo-HA-h*UVRAG* and pCI-neo-HA-h*ATG14* were purchased from Addgene (Cambridge, MA, USA).

Small interfering RNAs targeting BECN1 (si*BECN1*) were generated by GenePharma (Shanghai, People's Republic of China) and were transfected with DharmaFECT4 (ThermoScientific, T-2004-02). Lentiviral plasmid vectors encoding short hairpin RNAs (shRNAs) targeting ASPP2 or scramble shRNA were generated and designated as LV-sh*ASPP2* and LV-shNon, respectively. Further details are available in the Supplementary Material.

### Real-time PCR and western blotting

Isolation of total cellular RNA was carried out by using the NucleoSpin RNAII (MACHEREY-NAGEL, 740955), and first-strand cDNA was generated using the PrimeScript RT reagent kit (Takara, DRR037A). The cDNA sample was then measured by real-time PCR with an Applied Biosystems 7500 Real-Time PCR System as recommended by the manufacturer.

Total cell lysate was prepared as described before.^19^ The primers and antibodies used in this study are listed in the Supplementary Table S5 and Supplementary Table S6. The nuclear extracts from cells were prepared using NE-PER Nuclear and Cytoplasmic Extraction Reagents Kit (Pierce, 78833).

### Luciferase reporter assays

HCC (3 × 10^4^) cells were plated in 48-well plates and transfected with pGL3.0-enhancer (Promega) and full or various truncated *BECN1* promoter-luciferase constructs together with the pRL-TK in triplicate by X-tremeGENE HP DNA Transfection Reagent (Roche, 06366236001). We treated cells as indicated at 24 h after transfection, and then collected cells for performing the luciferase assays with the Dual Luciferase Reporter Assay System (Promega). Luciferase activities were calculated as fold induction compared with that in pGL3.0-enhancer. All bar diagrams are shown as the means±S.D.

### Animal studies

Six-week-old male athymic BALB/c nude mice were purchased from the Shanghai Experimental Animal Center of Chinese Academic of Sciences (Shanghai, People's Republic of China) and were maintained in specific pathogen-free conditions. Animal care and experimental protocols were conducted in accordance with the guidelines of Shanghai Medical Experimental Animal Care Commission. For *in vivo* treatment, HCC-LM3 cells (5 × 10^6^) infected with LV-shNon and LV-sh*ASPP2* (at a MOI of 50) were implanted subcutaneously into the flank of nude mice (six in each group, male BALB/c nu/nu). By day 7, tumors were well established in the mice with an average size of ~300 mm^3^. Mice were intraperitoneally given 5-FU (10 mg/kg body weight) daily for 5 days or chloroquine (45 mg/kg body weight) every 3 days for a total of six times. The mice were killed 30 days later, and the tumors and surrounding tissues were isolated for histopathology examination.

### Patient samples and immunohistochemical staining

One hundred and eighty-six primary HCC samples were obtained from patients who had undergone curative hepatic resection at Guangxi Cancer Hospital (Nanning, Guangxi, People's Republic of China). The expression of ASPP2 and BECN1 were analyzed with immunohistochemistry assay. Evaluation of immunostaining was independently performed by two experienced pathologists. Details can be found in the Supplementary Material.

### Statistic analysis

All statistical analyses were carried out using SPSS 16.0 for Windows software. The *χ*^2^ test was used to compare qualitative variables; quantitative variables were analyzed by two-tailed Student's *t-*test and Wilcoxon rank sum test. Kaplan–Meier analysis was used to determine the survival data. Data were presented as the mean±S.E.M. All statistical tests were two-sided, and *P*<0.05 was considered statistically significant.

Other Material and Methods are available in the Supplementary Material and Methods.
