# Supplementary material for: Downregulation of ASPP2 improves hepatocellular carcinoma cells survival via promoting BECN1-dependent autophagy initiation
Source: Cell Death Dis. 2016 Dec 8;7(12):e2512–. doi: 10.1038/cddis.2016.407 (PMC5260975; doi:10.1038/cddis.2016.407)
Supplement: Supplementary Material [file cddis2016407x1.docx]

**Supplementary Material**

**Materials and Methods**

**shRNA design and construction**

Three pairs of cDNA oligonucleotides were designed to target ASPP2 mRNA expression with web-based software from InvivoGen Inc. (San Diego, CA; http://www.sirnawizard.com/design.php) and Invitrogen (http://rnaidesigner.invitrogen.com/rnaiexpress/). After synthesized, these double-strand oligos were inserted to the vector pENTR/U6 (Invitrogen). The resulting plasmids were sequenced to ensure the shRNA construct targeted human ASPP2 expression or were scramble as design. Then, the plasmids were transfected into HCC-LM3 cells. Real-time PCR and Western blot were used to validate gene silencing efficiency of these shRNA plasmids 48 hours after transfection. The double-strand oligo DNAs with the silencing efficiency for ASPP2 were as follows: #1, 5’-GCTGAGGGAGAAAGAGAAGAA-3’; #2, 5’-CTTTCTTATCTAATCCTTA-3’; #3, 5’- CAACTAAATTACTGCCTTT -3’For negative control, we used scramble shRNA(5’-AATTCTCCGAACGTGTCACGT-3’).

**Lentivirial vector production and Lentivirus infection**

We generated lentiviral vectors encoding shRNAs using pLenti6/BLOCKiT-DEST (Invitrogen) recombined with pENTR-U6-shRNA plasmids by the Gateway cloning system. To produce lentivirus containing shRNA, HEK-293T cells were co-transfected with pLenti/BLOCKiT-DEST-shRNA plasmids and ViraPower Packaging Mix (Invitrogen) using Lipofectamine 2000 according to the manufacturer’s guidelines. Infectious lentiviruses were harvested at 48 and 72 h after transfection and filtered through 0.45μm PVDF filters. Recombinant lentiviruses were concentrated 100-fold by ultracentrifugation (2 h at 50,000 g). The virus-containing pellet was dissolved in DMEM, aliquoted and stored at -80 °C. The infectious titer was determined by counting the blue-stained colonies after crystal violet staining in 293 cells.

Lentiviral plasmid vectors encoding short hairpin RNAs (shRNAs) targeting ASPP2 or scramble shRNA were generated and designated as LV-sh*ASPP2* and LV-shNon, respectively. HCC-LM3 cells or HepG2 cells were infected with concentrated virus at a multiplicity of infection of 20 in the presence of 8 μg/ml polybrene (Sigma). Supernatant was removed after 24 h and replaced with complete culture medium. Seventy-two hours after infection, the transduced cells were confirmed by Real-time PCR and Western blotting. The effect of silencing of ASPP2 in HCC cells was shown in Supplementary Fig. 1.

**Autophagy analysis with GFP-LC3**

Autophagy was assessed by GFP microtubule-associated protein light chain 3 (GFP-LC3) redistribution[^1^](#_ENREF_1). For GFP-LC3 redistribution, HCC cells were transfected with a GFP-LC3 expression plasmid. Redistribution was detected after transfection using an inverted fluorescence microscope. The fraction of GFP-LC3-positive (>3 punctate staining sites per cell) cells was determined in three independent experiments. Ten random fields representing 200 cells were counted.

**Electron Microscopy**

Cells were washed with serum-free Dulbecco’s modified Eagle’s medium with a brief wash in prewarmed 0.1 M cacodyate buffer and then fixed with 2.5% glutaraldehyde with 0.1 M sodium cacodylate buffer for 1 h. The cells were washed, post-fixed with 2% osmium tetroxide, dehydrated with ascending grades of ethanol and propylene oxide, and embedded in LX-112 medium (Ladd). After polymerization, ultra-thin (90 nm) sections were cut with a diamond knife, collected on uncoated copper grids and stained with uranyl acetate (1%) and lead citrate (0.2%). The samples were examined with a HITACHI 7650 electron microscope operated at 100 Kv. For quantitative analysis of autophagy, the number of autophagic vesicles per viable cell was scored. For quantification of viable cells using electron micrographs, high-powered micrographs (8,000–10,000) of 20 single cells from multiple distinct low-powered fields were obtained from each specimen.

**Immunoﬂuorescence**

Cultured cells were washed 3 times with cold phosphate buffer saline (PBS), fixed with 4% paraformaldehyde, blocked and then incubated with indicated primary antibodies (see Supplementary Table 6) overnight at 4°C. After washed with PBS, cells were incubated with corresponding Alexa 488-conjugated and/or Alexa 555-conjugated secondary antibodies (Invitrogen) for 1 h at room temperature and then 4,6-diamidino-2-phenylindole (DAPI; Vector Laboratories Inc., Burlingame, CA) for 5 min. Representative images were captured with Olympus IX70.

**Chromatin-Immunoprecipitation Analysis**

Chromatin-immunoprecipitation (ChIP) analysis was performed by using the Chromatin immunoprecipitation Assay Kit (Millipore, 17-295). Antibodies used for ChIP were anti-RelA/p65 or anti-Flag antibody. DNA was purified with phenol/chloroform and a fraction was used as the PCR template to detect the presence of the promoter sequences between -33 to +155 of *BECN1* using specific primers: 5’-TGCTGCCGTCGTAGCGTCAC-3’ and 5’- CCCCCGATGCTCTTCACCTC-3’. We analyzed the amplification production by electrophoresis in 2% agarose.

**Immunoprecipitation Assay**

After different treatment, cells were incubated with lysis buffer (50 mM Tris HCl, 150 mM NaCl, 1% NP-40, 0.5% sodium deoxycholate, 0.1% SDS) plus protease inhibitors [1 mM PMSF (Sigma, P7626), Protease inhibitor cocktail tablets (Roche, 04693116001)]. Cells were lysed for 30 min on ice, and the centrifuged at 16000g for 20 min at 4°C. The cell lysates (1 mg protein) were incubated overnight with 2 μg of antibody, and the immune complexes precipitated with protein A/G agarose (Santa Cruz, sc-2003) for 3 hours at 4°C. Complexes were washed in lysis buffer (5×5 min). Immunoprecipitated proteins were analyzed by western blotting.

**PIK3C3 kinase assay**

The PIK3C3 kinase assay was performed with PI3K ELISA Pico Assay kit (Ecoslon, K-1000s) which quantifies the amount of ADP produced by the PI(3)KC3 reaction. Briefly, 30 μl PIK3C3 protein immunoprecipitated by anti-BECN1 antibody from HCC-LM3 lysates and 30 μl of 10 μM PI(4,5)P_2_ substrate were added into micro centrifuge tubes and incubated at 37°C for 2-3 hours. Then the 60 μl kinase reactions were stopped and transferred into 2 wells of the incubation plate for duplicate data points. PI(3,4,5)P_3_ detectors were added and incubated for 60 min at room temperature. After incubated with the secondary detector for 30 min, 100 μl TMB solution were added into plate, which was incubated for 30 min in dark, and intensity read absorbance at 450 nm on a plate reader. The kinase activity can be estimated by comparing the absorbance values from the wells containing enzyme reaction products to the values in the standard curve, which was prepared with the PI(3,4,5)P_3_ standards.

The analysis of foci formation of p40(phox)PX-EGFP fusion protein, which specifically binds to PtdIns(3)P produced by PIK3C3, could be used to assay PIK3C3 kinase activity. Cells were transfected with p40(phox)PX-EGFP for 24h and incubated in EBSS for an additional indicated time. The number of p40(phox)PX-EGFP-positive vesicle per cell was determined using an inverted fluorescence microscope.

**Plate colony formation and Anchorage-Independent Growth Assay**

Forty-eight hours after transfection, cells were dispersed into single-cell suspension which was prepared and inoculated in 100 mm dishes with a density of 5×10^3^ cells and maintained for 12 days. Afterwards, the colonies were stained with 1% crystal violet for 30s after fixation with 4% paraformaldehyhe for 5 minutes, and the colonies were counted. Each experiment was repeated in triplicate. The results presented are averages from three independent experiments.

For anchorage-independent growth assay, the cells in single-cell suspension were plated in 0.3% agarose over a 0.6% agarose bottom layer at a density of 500 cells per well in 24-well plates and incubated for 14 days. Finally, the numbers of cellular colonies greater than 200 μm in diameter were counted.

For blocking autophagy, 3-MA (10 mM) was used to pre-treat cells one hour in these experiments.

**MTS Assay**

Cells were cultured in 96-well (2500 cells per well) at 48 hours after infection with LV-shNon or LV-sh*ASPP2*. Then they were treated by 5-FU and VP16 for the indicated times. Cell growth was measured by MTS assay (Promega, G3580) in 96-well plates following the instructions of the manufacturer. Each experiment was done in triplicate. For blocking autophagy, 3-MA (10 mM) was used to pre-treat cells one hour in these experiments.

**Detection of Apoptosis**

Apoptotic cells were analyzed by the ApoDETECT Annexin V-FITC kit (Invitrogen, 33-1200) in vitro. Cells were cultured in six-well plates to 70–80% confluence, then incubated in EBSS or chemotherapy drugs at 37°C for the indicated times. 3-MA (10 mM) was used to block autophagy. Annexin V-FITC assay was used to measure apoptotic cells by flow cytometry according to the manufacturer’s instructions (Invitrogen). Briefly, cells were collected by trypsinization, washed with ice-cold phosphate-buffered saline (PBS) twice and resuspended in 300 μl 1x binding buffer containing 5 μl Annexin V and 5 μl PI for 30 minutes at 4°C in the dark. After incubation, at least 10,000 cells were measured on a BD FACSAria flow cytometer (Becton Dickinson). Results were expressed as the percentage of apoptotic cells at early stage (PI-negative and Annexin V-positive).

**TUNEL apoptosis analysis**

TUNEL assays on FFPE xenograft tumor tissue sections were done by DeadEnd Colorimetric TUNEL system (Promega) according to the manufacturer’s instructions. Apoptotic cells were observed under a microscope (Olympus, Tokyo, Japan). The apoptotic nuclei were stained as dark brown. Positive-labeled cells were counted in five randomly selected fields and expressed as percentage of total cells counted.

Briefly, sections were rehydrated, permeabilized and inactivated of endogenous peroxidases. Then the sections were incubated at 37 °C for 90 min with terminal deoxynuleotidyl trasferase (TdT) Labeling Reaction Mix (biotin-labeled) and covered with Stop Solution for termination of labeling reaction. Biotinylated nucleotides are detected using a streptavidin-horseradish peroxidase (HRP) conjugate. Diaminobenzidine reacts with the labeled tissues to generate an insoluble colored substrate at the conjugated sites. At last, sections were counterstained with methyl green in the morphological evaluation and characterization of normal and apoptotic cells.

**Patient samples**

One hundred and eighty-six primary HCC samples were obtained from patients who had undergone curative hepatic resection between 2003 and 2006 at Guangxi Cancer Hospital (Nanning, Guangxi, P.R.China). Patient’s consent and approval from Guangxi Cancer Hospital Ethics Committee were obtained in order to use these clinical materials for research purposes. The entering criteria of all patients were described as previously reported. Curative resection was defined as complete resection of all tumor nodules and the cut surface being free of cancer by histologic examination. The clinicopathologic features of the patients were summarized in Supplementary Table S1. Patient follow-up was completed on March 15, 2011. The median follow-up period was 41 months (range, 1–79 months). Overall survival (OS) was defined as the interval between the dates of surgery and death. Recurrence-free survival (RFS) was defined as the interval between the dates of surgery and recurrence; if recurrence was not diagnosed, patients were censored on the date of death or the last follow-up.

**Immunohistochemical staining**

The expression of ASPP2 and BECN1 were analyzed with EnVision system (Changdao Bio-tech) in formalin-fixed, paraffin-embedded sections of primary tumors. Briefly, the slides were dewaxed, hydrated and washed, and the endogenous peroxidase activity was quenched. After microwave antigen retrieval, slides were blocked and then incubated with the antibody against ASPP2 (Sigma-Aldrich, A4480) or BECN1 (Epitomics, T2026) overnight at 4°C. Subsequently, sections were rinsed and incubated with the working solution of horseradish peroxidase-labeled goat anti-rabbit for 60 minutes at 37°C. After rinse for three times, diaminobenzidine colorimetric reagent solution from Dako (Carpinteria, CA) was used. Counterstained by hematoxylin, the slides were dehydrated in graded alcohol and mounted. Negative controls were prepared in the absence of primary antibody.

Evaluation of immunostaining was independently performed by two experienced pathologists. The expression of ASPP2 and BECN1 were scored according to the signal intensity and distribution. Briefly, a mean percentage of high tumor cells was determined in at least five areas at ×400 magnification and assigned to one of the five following categories: 0, <5%; 1, 5-25%; 2, 25-50%; 3, 50-75% and 4, >75%. The intensity of immunostaining was scored as follows: 1, weak; 2, moderate and 3, intense. For tumors that showed heterogeneous staining, the predominant pattern was taken into account for scoring. The percentage of high tumor cells and the staining intensity were multiplied to produce a weighted score for each case. Tissues with immunohistochemical scoring ≤2 were considered as low, 3-12 as high.

**Statistic Analysis**

All statistical analyses were carried out using SPSS 16.0 for Windows software. The χ2 test was used to compare qualitative variables; quantitative variables were analyzed by 2-tailed Student’s t test and Wilcoxon rank sum test. Clinical variables included age, gender, HBV active status, α-fetoprotein, cirrhosis, tumor number, vascular invasion, tumor size and stage. Tumor stage was determined according to the American joint committee on cancer (AJCC) classification system. Kaplan-Meier analysis was used to determine the survival data. Overall survival was defined as the interval between surgery and death or between surgery and the last observation point. For surviving patients, the data were censored at the last follow-up. Recurrence-free survival was defined as the interval between the date of surgery and the date of diagnosis of any type of relapse. Difference in survival between groups was evaluated by the log-rank test. Univariate and multivariate analyses were based on the Cox proportional hazards regression model. Data were presented as the mean ± SEM. All statistical tests were two-sided, and *P*<0.05 was considered statistically significant.

**Supplementary Figures**

Supplementary Figure 1

**
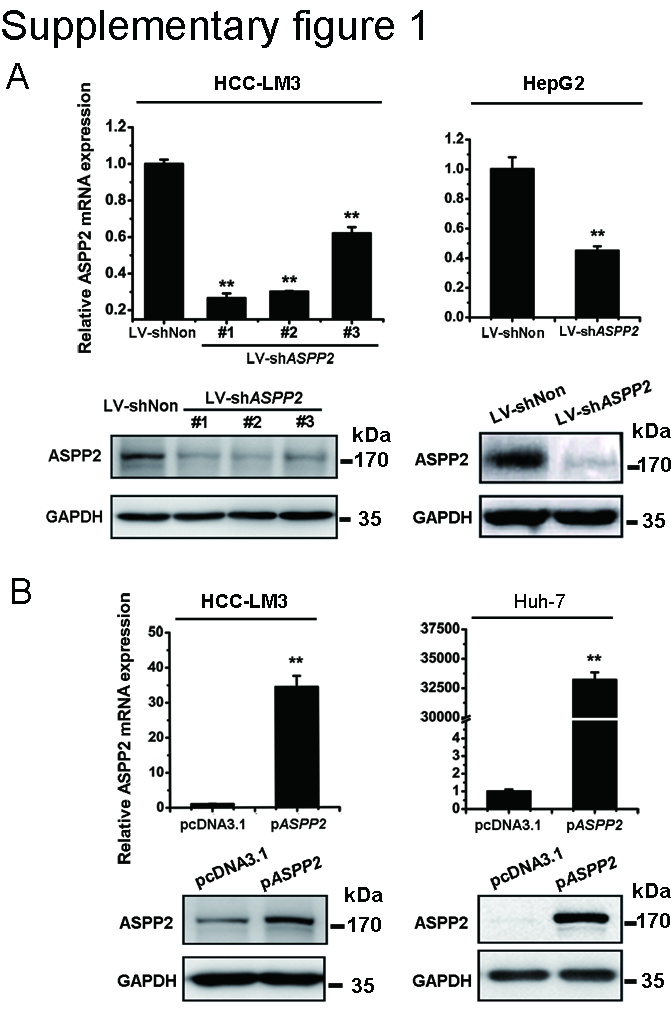
**

Supplementary Figure 1. Effect of silencing and overexpression of ASPP2 in HCC cells. (A) HCC cells were infected with lentiviruses encoding shRNA against ASPP2 for 72 hours. HCC-LM3 was infected with LV-shNon or LV-sh*ASPP2* (#1, #2 or #3), which targeted to 3 different regions of ASPP2 mRNA (left). As results shown, LV-sh*ASPP2* #1, refered to as LV-sh*ASPP2* in other parts of the paper, was used to knockdown the expression of ASPP2 in HepG2 (right). (B) HCC-LM3 (left) and Huh7 (right) cells were transfected with pcDNA3.1 or p*ASPP2* for 48 hours. The expression of ASPP2 was detected by qRT-PCR and western blot. Data represented the mean ± SD *, *P* < 0.05; **, *P* < 0.01.

Supplementary Figure 2


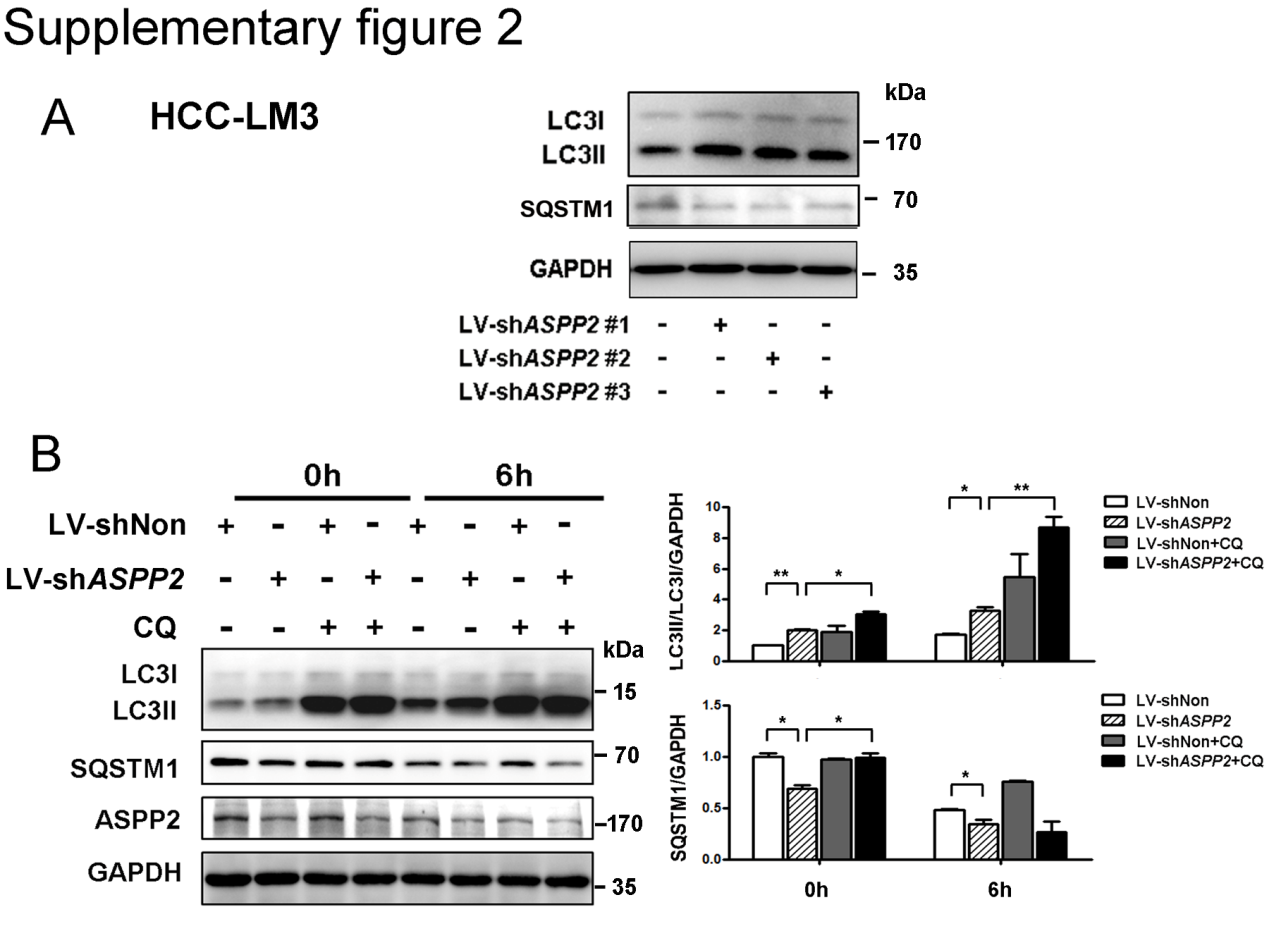


Supplementary Figure 2. Induction of autophagy in ASPP2 silencing HCC-LM3 cells. (A) HCC-LM3 was infected with LV-shNon or LV-sh*ASPP2* (#1, #2 or #3) for 72h before incubation in EBSS for 6h. Cell lysates were subjected to western blotting. (B) Western blot shows LC3 and SQSTM1 expression in lysates from LV-shNon or LV-sh*ASPP2* HCC-LM3 cells treated with EBSS for indicated time with or without CQ. Band intensity was quantified using ImageJ. Data were obtained from 3 independent experiments. Data represented the mean ± SD from triplicate experiments. (*, *P* < 0.05; **, *P* < 0.01). EBSS, serum free Earle’s Balanced Salt Solution medium; CQ, chloroquine.

Supplementary Figure 3


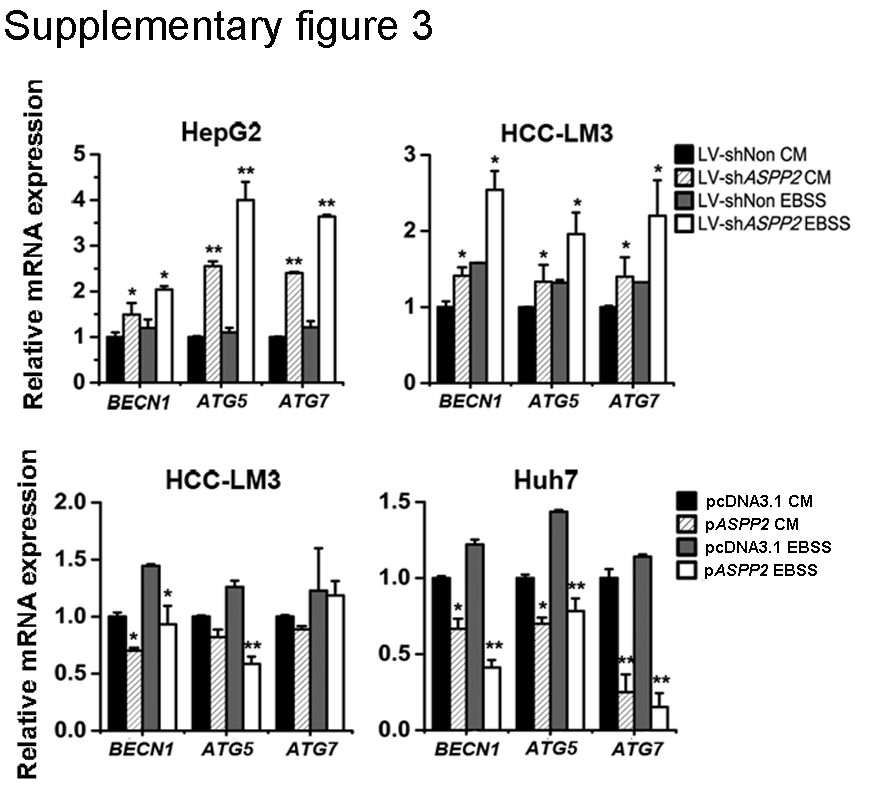


Supplementary Figure 3. ASPP2 affects the mRNA level of *BECN1*, *ATG5* and *ATG7* in HCC cells. HCC-LM3 and HepG2 cells were infected with LV-shNon or LV-sh*ASPP2* for 72h (upper); HCC-LM3 and Huh7were transfected with pcDNA3.1 or p*ASPP*2 for 48h (lower). After cultured in normal condition or EBSS for 6h, cells were performed for the analysis *BECN1,* *ATG5* and *ATG7* mRNA level with Quantitative PCR. Data represented the mean ± SD *, *P* < 0.05; **, *P* < 0.01. CM, complete medium; EBSS, serum free Earle’s Balanced Salt Solution medium.

Supplementary Figure 4


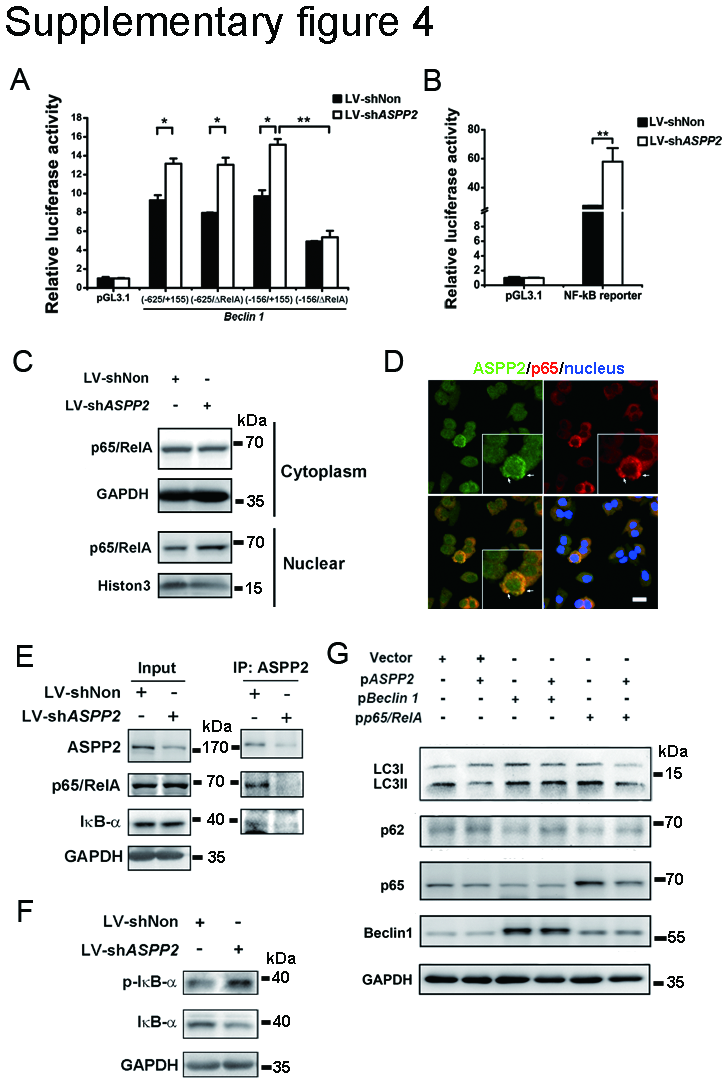


Supplementary Figure 4. Down-regulation of ASPP2 promotes NF-κB/p65-dependent transactivation of BECN1. (A) HCC-LM3 cells infected with LV-shNon or LV-sh*ASPP2* were transfected with BECN1 (-625/+155) and (-156/+155)-luc or the promoter containing p65/RelA consensus binding site mutation (cggggtttca→aaattgaaga, BECN1-625/ΔRelA) and (gggaagtcgc→aagatgaagc, BECN1-156/ΔRelA), and luciferase activities were measured at 48h post-transfection followed by 6h EBSS treatment. Data are shown as the means ± SD from triplicate experiments. (B) HCC-LM3 cells infected with LV-shNon or LV-sh*ASPP2* were transfected with NF-κB driven-luciferase construct. (C) HCC-LM3 cells were infected with LV-shNon or LV-sh*ASPP2* for 72h. After treatment of EBSS for 6h, the nuclear and cytoplasm extracts were immunoblotted with the indicated antibodies. (D) ASPP2 colocalized with p65/RelA in cytoplasm under starvation environment. After HCC-LM3 cells were treated with EBSS for 6h, location of endogenous ASPP2 and p65/RelA was analyzed by double immunofluorescent (IF) staining. Scale bars: 30 μm. (E) ASPP2 interacts with p65/RelA and IκBα. ASPP2 were silenced by LV-sh*ASPP2* infection for 72h in HCC-LM3. Endogenous ASPP2-p65/RelA and ASPP2-IκBα complexes were immunoprecipitated with anti-ASPP2 and analyzed for co-immunoprecipiatation of ASPP2-p65/RelA and ASPP2-IκBα conjugates (IP). (F) Downregulation of ASPP2 promotes phosphorylation of IkBα. ASPP2 was silenced by LV-sh*ASPP2* infection for 72h in HCC-LM3, which were then incubated in EBSS for 8h. Cell lysates were subjected to western blotting. (G) HCC-LM3 cells were co-transfected p*ASPP2* and p*BECN1* or p*p65/RelA* for 48h, following incubation with EBSS for 6h. Cell lysates were analyzed by western blotting.

**Supplementary tables**

**Supplementary Table 1. The clinicopathologic characteristics of 186 cases of HCC**

| **Variables** | **No.of patients(%)** |
| --- | --- |
| Gender |  |
| Male | 167 (90) |
| Female | 19(10) |
| Age (year) |  |
| ＜50 | 111 (60) |
| ≥50 | 75(40) |
| HBsAg |  |
| Positive | 176 (95) |
| Negative | 10 (5) |
| AFP (ng/ml) |  |
| ≤400 | 85 (42) |
| ＞400 | 101 (58) |
| Cirrhosis |  |
| － | 62 (33) |
| ＋ | 124 (67) |
| Tumor volume (cm3) |  |
| ＜5 | 66(35) |
| ≥5 | 120 (65) |
| Vascular invasion |  |
| No | 165 (89) |
| Yes | 21(11) |
| Tumor number |  |
| Single | 168(90) |
| Multiple | 18 (10) |
| AJCC stage |  |
| Ⅰ-Ⅱ | 111(60) |
| Ⅲ-Ⅳ | 75(40) |
| Recurrence |  |
| － | 42 (23) |
| ＋ | 144 (77) |

Abbreviations: AFP, alpha fetoprotein; HBsAg, hepatitis B surface antigen; AJCC, American Joint Committee on Cancer

**Supplementary Table 2. The associations of ASPP2 and BECN1 expression with clinicopathologic characteristics in 186 patients with HCC**

|  | **Whole study(n=186)** | | | **ASPP2 low group(n=64)** | | | **ASPP2 high group(n=122)** | | |
| --- | --- | --- | --- | --- | --- | --- | --- | --- | --- |
|  | **BECN1 expression** | | ***P*** | **BECN1 expression** | | ***P*** | **BECN1 expression** | | ***P*** |
|  | **Low** | **high** |  | **low** | **high** |  | **low** | **high** |  |
|  | **(n=94)** | **(n=92)** |  | **(n=21)** | **(n=43)** |  | **(n=73)** | **(n=49)** |  |
| **Sex** |  |  |  |  |  |  |  |  |  |
| male | 84 | 83 | 0.52 | 1 | 5 | 0.7 | 9 | 4 | 0.7 |
| Female | 10 | 9 |  | 20 | 38 |  | 64 | 45 |  |
| **Age(years)** |  |  |  |  |  |  |  |  |  |
| <50 | 50 | 61 | 0.047 | 16 | 27 | 0.284 | 34 | 34 | 0.013 |
| ≥50 | 44 | 31 |  | 5 | 16 |  | 39 | 15 |  |
| **HBsAg** |  |  |  |  |  |  |  |  |  |
| negative | 4 | 6 | 0.36 | 1 | 4 | 0.889 | 3 | 2 | 1.00 |
| positive | 90 | 86 |  | 20 | 39 |  | 70 | 47 |  |
| **AFP(ng/ml)** |  |  |  |  |  |  |  |  |  |
| ≤20 | 41 | 44 | 0.334 | 7 | 17 | 0.63 | 34 | 27 | 0.356 |
| >20 | 53 | 48 |  | 14 | 26 |  | 39 | 22 |  |
| **Tumor volume(cm3)** |  |  |  |  |  |  |  |  |  |
| ≤5 | 43 | 23 | 0.002 | 10 | 7 | 0.008 | 33 | 16 | 0.166 |
| >5 | 51 | 69 |  | 11 | 36 |  | 40 | 33 |  |
| **cirrhosis** |  |  |  |  |  |  |  |  |  |
| - | 36 | 26 | 0.097 | 12 | 11 | 0.013 | 24 | 15 | 0.793 |
| + | 58 | 66 |  | 9 | 32 |  | 49 | 34 |  |
| **Vascular invasion** |  |  |  |  |  |  |  |  |  |
| NO | 85 | 80 | 0.455 | 20 | 38 | 0.669 | 65 | 42 | 0.583 |
| YES | 9 | 12 |  | 1 | 5 |  | 8 | 7 |  |
| **Tumor number** |  |  |  |  |  |  |  |  |  |
| Single | 84 | 84 | 0.654 | 20 | 37 | 0.497 | 64 | 47 | 0.216 |
| Multiple | 10 | 8 |  | 1 | 6 |  | 9 | 2 |  |
| **AJCC Stage** |  |  |  |  |  |  |  |  |  |
| StageⅠ-Ⅱ | 57 | 54 | 0.452 | 10 | 20 | 0.934 | 47 | 34 | 0.566 |
| Stage Ⅲ-Ⅳ | 37 | 38 |  | 11 | 23 |  | 26 | 15 |  |
| **Recurrence time(months)** |  |  |  | 85 |  |  |  |  |  |
| ≤6 | 16 | 26 | 0.048 | 3 | 19 | 0.037 | 13 | 7 | 0.167 |
| >6 | 78 | 66 |  | 18 | 24 |  | 59 | 15 |  |

NOTE:*P* values are two-tailed and based on the Pearson χ^2^ test.

**Supplementary Table 3. Univariate analyses of factors associated with recurrence-free survival and overall survival**

|  | **RFS** | | **OS** | | |
| --- | --- | --- | --- | --- | --- |
| **Variables** | **Hazard ratio (95% CI)** | ***P*** | **Hazard Ratio (95% CI)** | ***P*** | |
| **Gender (male vs female)** | 0.798 (0.399-1.596) | 0.524 | 0.556 (0.276-2.1.119) | 0.10 |  |
| **Age, y ( ≥50 vs<50)** | 0.806 (0.512-1.270) | 0.353 | 0.761 (0.481-1.203) | 0.242 |  |
| **HbsAg ( positive vs negative)** | 2.098 (0.498-8.847) | 0.313 | 2.559 (0.615-10.645) | 0.197 |  |
| **AFP, ng/ml (>400 vs≤400)** | 0.941 (0.608-1.456) | 0.784 | 0.960 (0.619-1.489) | 0.856 |  |
| **Cirrhosis ( yes vs no)** | 1.401 (0.870-2.255) | 0.165 | 1.171 (0.724-1.894) | 0.519 |  |
| **Tumor size, cm (≥5vs<5)** | 1.070 (0.649-1.764) | 0.790 | 1.233 (0.745-2.039) | 0.415 |  |
| **Tumor number( multiple vs single)** | 1.550 (0.815-2.949) | 0.181 | 2.207 (1.172-4.157) | 0.014 |  |
| **AJCC stage (Ⅲ-ⅣvsⅠ-Ⅱ)** | 2.502 (1.504-4.164) | <0.001 | 1.409 (0.837-2.230) | 0.197 |  |
| **Vascular invasion ( yes vs no)** | 1.212 (0.655-2.243) | 0.540 | 1.383(0.759-2.520) | 0.290 |  |
| **ASPP2 ( high vs low)** | 0.603 (0.393-0.926) | 0.021 | 0.528 (0.337-0.828) | 0.005 |  |
| **BECN1 ( high vs low)** | 1.574 (1.023-2.422) | 0.039 | 0.495 (0.321-0.763) | 0.001 |  |

**Supplementary Table 4. Multivariate analyses of factors associated with recurrence-free survival and overall survival**

|  | **Hazard ratio (95% CI)** | ***P*** |
| --- | --- | --- |
| **RFS** |  |  |
| **AJCC stage (Ⅲ-ⅣvsⅠ-Ⅱ)** | 1.964 (1.285-3.000) | 0.002 |
| **ASPP2 (high vs low)** | 0.623 (0.405-0.960) | 0.032 |
| **BECN1 (high vs low)** | 1.669(1.090-2.555) | 0.018 |
| **OS** |  |  |
| **Tumor number(multiple vs single)** | 2.645(1.456-4.805) | 0.001 |
| **ASPP2 (high vs low)**  **BECN1 (high vs low)** | 0.457(0.297-0.703)  1.575(1.032-2.405) | <0.001  0.035 |

Multivariate analysis, Cox proportional hazards regression model.

Variables were adopted for their prognostic significance by univariate analysis and no obvious correlation between each other.

**Supplementary Table 5. Primers used in this study**

| **Primers for Real-time PCR** | | |
| --- | --- | --- |
| **Protein** | | **Sequence (5’→3’)** |
| BECN1 | F | AGCTGCCGTTATACTGTTCTG |
|  | R | ACTGCCTCCTGTGTCTTCAATCTT |
| ASPP2 | F | GAAGACTCGGTGAGCATGCG |
|  | R | GCGATACGCTCTGAGCCAGT |
| Atg5 | F | TGGGCCATCAATCGGAAACTC |
|  | R | TGCAGCCACAGGACGAAACAG |
| Atg7β | F | GCAAGCCCGCAGAGATGTGGA |
|  | R | GCAGCAATGACGGCAGGAAGC |
| β-actin | F | CGTGGACATCCGTAAAGACC |
|  | R | ACATCTGCTGGAAGGTGGAC |

**Supplementary Table 6. Antibodies used in this study**

| **Antibodies used in the research** | | |
| --- | --- | --- |
| **Protein** | **Usage** | **Antibody** |
| ASPP2 | WB | A4480, Sigma-Aldrich |
| GAPDH | WB | KC5G5, KangChen Bio-tech |
| LC3B | WB | 2775, Cell signaling technology |
| SQSTM1/p62 | WB | 8025, Cell signaling technology |
| BECN1 | WB | 3738, Cell signaling technology |
| BECN1 | IP | 4122, Cell signaling technology |
| BECN1 | IHC | 2026, Epitomics |
| NF-κB p65 | WB | 8242, Cell signaling technology |
| NF-κB p65 | WB | 6956, Cell signaling technology |
| Histone H3 | WB | 4499, Cell signaling technology |
| PI3 Kinase Class III | WB | 4263, Cell signaling technology |
| UVRAG | WB | 5320, Cell signaling technology |
| Atg14 | WB | 5504, Cell signaling technology |
| Rubicon | WB | ab92388, Abcam |
| Bcl-2 | WB | 2870, Cell signaling technology |
| HA-Tag | WB, Co-IP | 3724, Cell signaling technology |
| IκBα | IF | 4814, Cell signaling technology |
| IκBβ | IF | Ab7547, Abcam |
| FLAG-Tag | WB, Co-IP | 9272, Cell signaling technology |
| V5-Tag | WB, Co-IP | V8137, Sigma-Aldrich |
| mouse normal IgG | WB, Co-IP | sc-2025, Santa Cruz |
| rabbit normal IgG | WB, Co-IP | sc-2027, Santa Cruz |

Abbreviations: WB, Western blotting; IHC, immunohisochemistry.

**Reference**

1 Klionsky DJ, Abdelmohsen K, Abe A, Abedin MJ, Abeliovich H, Acevedo Arozena A *et al*. Guidelines for the use and interpretation of assays for monitoring autophagy (3rd edition). Autophagy 2016; 12: 1-222.
